# Ostrowski type inequalities involving conformable fractional integrals

**DOI:** 10.1186/s13660-018-1664-4

**Published:** 2018-04-03

**Authors:** Muhammad Adil Khan, Sumbel Begum, Yousaf Khurshid, Yu-Ming Chu

**Affiliations:** 1 College of Science, Hunan City University, Yiyang, China; 2 Department of Mathematics, University of Peshawar, Peshawar, Pakistan; 3 Department of Mathematics, Huzhou University, Huzhou, China

**Keywords:** 26D15, 26A51, 26A33, 26E60, Ostrowski inequality, Conformable derivative, Conformable integral, Arithmetic mean, Generalized logarithmic mean

## Abstract

In the article, we establish several Ostrowski type inequalities involving the conformable fractional integrals. As applications, we find new inequalities for the arithmetic and generalized logarithmic means.

## Introduction

Let $I\subseteq\mathbb{R}$ be an interval and $I^{\circ}$ the interior of *I*. Then the classical Ostrowski inequality [1] states that a real-valued function $f: I\rightarrow\mathbb{R}$ satisfies the inequality

$$ \biggl\vert f(x)-\frac{1}{a_{2}-a_{1}} \int_{a_{1}}^{a_{2}}f(x)\,dx \biggr\vert \leq \biggl[ \frac{1}{4}+\frac{ (x-\frac{a_{1}+a_{2}}{2} )^{2}}{(a_{2}-a_{1})^{2}} \biggr] (a_{2}-a_{1})\big\| f^{\prime}\big\| _{\infty} $$

with the best possible constant $1/4$ if $a_{1}, a_{2}\in I^{\circ}$ with $a_{1}< a_{2}$ and $|f^{\prime}(x)|\leq M$ for all $x\in[a_{1}, a_{2}]$.

Recently, the Ostrowski inequality has attracted the attention of many researchers, many remarkable generalizations, extensions, variants and applications can be found in the literature [2–24].

Let $0<\alpha\leq1$ and *g* be a real-valued function defined on $[0, \infty)$. Then the (conformable) fractional derivative $D_{\alpha }(g)(t)$ [23] of order *α* of *g* at $t>0$ is defined by

$$ D_{\alpha}(g) (t)=\lim_{\epsilon\rightarrow0}\frac{g(t+\epsilon t^{1-\alpha})-g(t)}{\epsilon}. $$

*g* is said to be *α*-differentiable if the conformable fractional derivative of order *α* of *g* exists. In what follows, we write $g^{\alpha}(t)$ or $\frac{d_{\alpha}}{d_{\alpha}t}(g)$ for $D_{\alpha }(g)(t)$ to denote the conformable fractional derivative of order *α* of *g*. The conformable fractional derivative at 0 is defined as $g^{\alpha}(0)=\lim_{t\rightarrow0^{+}}g^{\alpha}(t)$.

Let $\alpha\in(0, 1]$ and $0\leq a< b$. Then the function $h: [a, b]\rightarrow\mathbb{R}$ is said to be *α*-fractional integrable on $[a, b]$ if the integral

$$ \int_{a}^{b}h(x)\,d_{\alpha}x:= \int_{a}^{b}h(x)x^{\alpha-1}\,dx $$

exists and is finite. All *α*-fractional integrable functions on $[a, b]$ are denoted by $L_{\alpha}^{1}([a, b])$.

### Remark 1.1

Note that the relation between the Riemann integral and the conformable fractional integral is given by

$$ I_{\alpha}^{a}(h) (t)=I_{1}^{a} \bigl(t^{\alpha-1}h\bigr)= \int_{a}^{t}\frac {h(x)}{x^{1-\alpha}}\,dx. $$

Let $\alpha\in(0, 1]$ and *f*, *g* be *α*-differentiable at $t>0$. Then it is well known that

$$ (1) \quad\frac{d_{\alpha}}{d_{\alpha}t}\bigl(t^{n}\bigr)=nt^{n-\alpha} $$

for all $n\in\mathbb{R}$;

$$ (2) \quad\frac{d_{\alpha}}{d_{\alpha}t}(c)=0 $$

for all constant $c\in\mathbb{R}$;

$$ (3) \quad\frac{d_{\alpha}}{d_{\alpha}t}\bigl(af(t)+bg(t)\bigr)=a\frac{d_{\alpha }}{d_{\alpha}t}\bigl(f(t) \bigr)+b\frac{d_{\alpha}}{d_{\alpha}t}\bigl(g(t)\bigr) $$

for all $a, b\in\mathbb{R}$;

$$\begin{gathered} (4) \quad\frac{d_{\alpha}}{d_{\alpha}t}\bigl(f(t)g(t)\bigr)=f(t)\frac{d_{\alpha }}{d_{\alpha}t}\bigl(g(t) \bigr)+g(t)\frac{d_{\alpha}}{d_{\alpha}t}\bigl(f(t)\bigr); \\ (5) \quad\frac{d_{\alpha}}{d_{\alpha}t} \biggl(\frac{f(t)}{g(t)} \biggr) =\frac{g(t)\frac{d_{\alpha}}{d_{\alpha}t}(f(t))-f(t)\frac{d_{\alpha }}{d_{\alpha}t}(g(t))}{g^{2}(t)}; \\(6) \quad\frac{d_{\alpha}}{d_{\alpha}t}\bigl(f\bigl(g(t)\bigr)\bigr)=f^{\prime} \bigl(g(t)\bigr)\frac {d_{\alpha}}{d_{\alpha}t}\bigl(g(t)\bigr),\end{gathered} $$

if *f* is differentiable at $g(t)$.

The main purpose of the article is to find the Ostrowski type inequalities involving the conformable fractional integrals and give their applications in certain bivariate means.

## Main results

### Lemma 2.1

*Let* $0<\alpha\leq1$, $0\leq a_{1}< a_{2}$ *and* $h: [a_{1}, a_{2}]\rightarrow\mathbb{R}$ *be an* *α*-*fractional differentiable function*. *Then the identity*

$$ \begin{aligned} h(x)-\frac{\alpha}{a_{2}^{\alpha}-a_{1}^{\alpha}} \int _{a_{1}}^{a_{2}}h(s)\,d_{\alpha}s ={}&\frac{x-a_{1}}{a_{2}^{\alpha}-a_{1}^{\alpha}} \int_{0}^{1} \bigl[\bigl((1-t)a_{1}+tx \bigr)^{2\alpha-1}-a_{1}^{\alpha}\bigl((1-t)a_{1}+tx \bigr)^{\alpha -1} \bigr] \\ &\times D_{\alpha}(h) \bigl((1-t)a_{1}+tx \bigr)t^{1-\alpha}\,d_{\alpha}t \\&+\frac{a_{2}-x}{a_{2}^{\alpha}-a_{1}^{\alpha}} \int_{0}^{1} \bigl[\bigl((1-t)a_{2}+tx \bigr)^{2\alpha-1}-a_{2}^{\alpha}\bigl((1-t)a_{2}+tx \bigr)^{\alpha -1} \bigr] \\ &\times D_{\alpha}(h) \bigl((1-t)a_{2}+tx \bigr)t^{1-\alpha}\,d_{\alpha}t\end{aligned} $$

*holds if* $D_{\alpha}(h)\in L_{\alpha}^{1}([a_{1}, a_{2}])$.

### Proof

It follows from integration by parts that

$$\begin{gathered} \frac{x-a_{1}}{a_{2}^{\alpha}-a_{1}^{\alpha}} \int_{0}^{1} \bigl[\bigl((1-t)a_{1}+tx \bigr)^{2\alpha-1}-a_{1}^{\alpha}\bigl((1-t)a_{1}+tx \bigr)^{\alpha -1} \bigr] D_{\alpha}(h) \bigl((1-t)a_{1}+tx \bigr)t^{1-\alpha}\,d_{\alpha}t \\\qquad{}+\frac{a_{2}-x}{a_{2}^{\alpha}-a_{1}^{\alpha}} \int_{0}^{1} \bigl[\bigl((1-t)a_{2}+tx \bigr)^{2\alpha-1}-a_{2}^{\alpha}\bigl((1-t)a_{2}+tx \bigr)^{\alpha -1} \bigr]\\ \qquad{}\times D_{\alpha}(h) \bigl((1-t)a_{2}+tx \bigr)t^{1-\alpha}\,d_{\alpha}t \\\quad=\frac{x-a_{1}}{a_{2}^{\alpha}-a_{1}^{\alpha}} \int_{0}^{1} \bigl[\bigl((1-t)a_{1}+tx \bigr)^{2\alpha-1}-a_{1}^{\alpha}\bigl((1-t)a_{1}+tx \bigr)^{\alpha -1} \bigr] D_{\alpha}(h) \bigl((1-t)a_{1}+tx \bigr)\,dt \\\qquad{}+\frac{a_{2}-x}{a_{2}^{\alpha}-a_{1}^{\alpha}} \int_{0}^{1} \bigl[\bigl((1-t)a_{2}+tx \bigr)^{2\alpha-1}-a_{2}^{\alpha}\bigl((1-t)a_{2}+tx \bigr)^{\alpha -1} \bigr]\\ \qquad{}\times D_{\alpha}(h) \bigl((1-t)a_{2}+tx \bigr)\,dt \\\quad=\frac{x-a_{1}}{a_{2}^{\alpha}-a_{1}^{\alpha}} \int _{0}^{1}\bigl[\bigl((1-t)a_{1}+tx \bigr)^{\alpha}-a_{1}^{\alpha}\bigr]h^{\prime} \bigl((1-t)a_{1}+tx\bigr)\,dt \\\qquad{}+\frac{a_{2}-x}{a_{2}^{\alpha}-a_{1}^{\alpha}} \int _{0}^{1}\bigl[\bigl((1-t)a_{2}+tx \bigr)^{\alpha}-a_{2}^{\alpha}\bigr]h^{\prime} \bigl((1-t)a_{2}+tx\bigr)\,dt \\\quad=\frac{x-a_{1}}{a_{2}^{\alpha}-a_{1}^{\alpha}} \bigl(\bigl((1-t)a_{1}+tx\bigr)^{\alpha}-a_{1}^{\alpha} \bigr) \frac{h((1-t)a_{1}+tx)}{x-a_{1}} \bigg|_{0}^{1} \\\qquad{}-\frac{x-a_{1}}{a_{2}^{\alpha}-a_{1}^{\alpha}} \int_{0}^{1}\alpha \bigl((1-t)a_{1}+tx \bigr)^{\alpha-1}h\bigl((1-t)a_{1}+tx\bigr)\,dt \\\qquad{}+\frac{a_{2}-x}{a_{2}^{\alpha}-a_{1}^{\alpha}} \bigl(\bigl((1-t)a_{2}+tx\bigr)^{\alpha}-a_{2}^{\alpha} \bigr) \frac{h((1-t)a_{2}+tx)}{x-a_{2}} \bigg|_{0}^{1} \\\qquad{}-\frac{a_{2}-x}{a_{2}^{\alpha}-a_{1}^{\alpha}} \int_{0}^{1}\alpha \bigl((1-t)a_{2}+tx \bigr)^{\alpha-1}h\bigl((1-t)a_{2}+tx\bigr)\,dt \\\quad=\frac{x-a_{1}}{a_{2}^{\alpha}-a_{1}^{\alpha}} \biggl(\frac{x^{\alpha }-a_{1}^{\alpha}}{x-a_{1}}h(x) -\frac{\alpha}{x-a_{1}} \int_{a_{1}}^{x}h(s)\,d_{\alpha}s \biggr) \\\qquad{}+\frac{a_{2}-x}{a_{2}^{\alpha}-a_{1}^{\alpha}} \biggl(\frac{a_{2}^{\alpha }-x^{\alpha}}{a_{2}-x}h(x) -\frac{\alpha}{a_{2}-x} \int_{x}^{a_{2}}h(s)\,d_{\alpha}s \biggr) \\\quad=h(x)-\frac{\alpha}{a_{2}^{\alpha}-a_{1}^{\alpha}} \int _{a_{1}}^{a_{2}}h(s)\,d_{\alpha}s.\end{gathered} $$

 □

### Theorem 2.2

*Let* $0<\alpha\leq1$, $0\leq a_{1}< a_{2}$, $h: [a_{1}, a_{2}]\rightarrow\mathbb{R}$ *be an* *α*-*fractional differentiable function and* $D_{\alpha}(h)\in L_{\alpha}^{1}([a_{1}, a_{2}])$. *Then the inequality*

$$ \biggl\vert h(x)-\frac{\alpha}{a_{2}^{\alpha}-a_{1}^{\alpha}} \int _{a_{1}}^{a_{2}}h(s)\,d_{\alpha}s \biggr\vert \leq \frac{x-a_{1}}{a_{2}^{\alpha}-a_{1}^{\alpha}}\triangle_{1}+\frac {a_{2}-x}{a_{2}^{\alpha}-a_{1}^{\alpha}}\triangle_{2} $$

*holds if* $|h^{\prime}(x)|$ *is convex*, *where*

$$\begin{gathered}\begin{aligned} \triangle_{1}={}&\frac{1}{6}a_{1}^{\alpha-1}x\big|h^{\prime}(a_{1})\big| +\frac{1}{12}x^{\alpha-1}a_{1}\big|h^{\prime}(a_{1})\big|+ \frac {1}{12}x\big|h^{\prime}(a_{1})\big|-\frac{1}{4}a_{1}^{\alpha}\big|h^{\prime}(a_{1})\big| \\&+\frac{1}{12}a_{1}\big|h^{\prime}(x)\big|+\frac{1}{12}x^{\alpha -1}a_{1}\big|h^{\prime}(x)\big| +\frac{1}{4}x\big|h^{\prime}(x)\big|-\frac{1}{2}a_{1}^{\alpha}\big|h^{\prime}(x)\big|,\end{aligned} \\\triangle_{2}=\frac{1}{6}a_{2}^{\alpha}\big|h^{\prime}(a_{2})\big|- \frac {1}{6}x^{\alpha}\big|h^{\prime}(a_{2})\big|+ \frac{1}{3}a_{2}^{\alpha}\big|h^{\prime}(x)\big| - \frac{1}{3}x^{\alpha}\big|h^{\prime}(x)\big|.\end{gathered} $$

### Proof

Let $y>0$, $\varphi_{1}(y)=y^{\alpha-1}$ and $\varphi_{2}(y)=-y^{\alpha }$. Then we clearly see that the functions $\varphi_{1}$ and $\varphi _{2}$ both are convex. It follows from Lemma 2.1 and the convexity of $\varphi_{1}$, $\varphi_{2}$ and $|h'|$ that

$$\begin{gathered} \biggl\vert h(x)-\frac{\alpha}{a_{2}^{\alpha}-a_{1}^{\alpha}} \int _{a_{1}}^{a_{2}}h(s)\,d_{\alpha}s \biggr\vert \\ \quad\leq\frac{x-a_{1}}{a_{2}^{\alpha}-a_{1}^{\alpha}} \int_{0}^{1} \bigl(\bigl((1-t)a_{1}+tx \bigr)^{\alpha}-a_{1}^{\alpha} \bigr)\big|h' \bigl((1-t)a_{1}+tx\bigr)\big|\,dt \\ \qquad{}+\frac{a_{2}-x}{a_{2}^{\alpha}-a_{1}^{\alpha}} \int_{0}^{1} \bigl(a_{2}^{\alpha }- \bigl((1-t)a_{2}+tx\bigr)^{\alpha} \bigr)\big|h' \bigl((1-t)a_{2}+tx\bigr)\big|\,dt \\ \quad\leq\frac{x-a_{1}}{a_{2}^{\alpha}-a_{1}^{\alpha}} \int_{0}^{1} \bigl(\bigl((1-t)a_{1}+tx \bigr)^{\alpha-1}\bigl((1-t)a_{1}+tx\bigr)-a_{1}^{\alpha} \bigr)\big|h'\bigl((1-t)a_{1}+tx\bigr)\big|\,dt \\ \qquad{}+\frac{a_{2}-x}{a_{2}^{\alpha}-a_{1}^{\alpha}} \int_{0}^{1} \bigl(a_{2}^{\alpha }- \bigl((1-t)a_{2}^{\alpha}+tx^{\alpha}\bigr) \bigr)\big|h'\bigl((1-t)a_{2}+tx\bigr)\big|\,dt \\ \quad\leq\frac{x-a_{1}}{a_{2}^{\alpha}-a_{1}^{\alpha}} \int_{0}^{1} \bigl(\bigl((1-t)a_{1}^{\alpha-1}+tx^{\alpha-1} \bigr) \bigl((1-t)a_{1}+tx\bigr)-a_{1}^{\alpha} \bigr)\big|h'\bigl((1-t)a_{1}+tx\bigr)\big|\,dt \\ \qquad{}+\frac{a_{2}-x}{a_{2}^{\alpha}-a_{1}^{\alpha}} \int_{0}^{1} \bigl(a_{2}^{\alpha}- \bigl((1-t)a_{2}^{\alpha}+tx^{\alpha} \bigr) \bigr)\big|h'\bigl((1-t)a_{2}+tx\bigr)\big|\,dt \\ \quad\leq\frac{x-a_{1}}{a_{2}^{\alpha}-a_{1}^{\alpha}} \int_{0}^{1} \bigl(\bigl((1-t)a_{1}^{\alpha-1}+tx^{\alpha-1} \bigr) \bigl((1-t)a_{1}+tx\bigr)-a_{1}^{\alpha} \bigr)\\ \qquad{}\times \bigl[(1-t)\big|h'(a_{1})\big|+t\big|h'(x)\big| \bigr]\,dt \\ \qquad{}+\frac{a_{2}-x}{a_{2}^{\alpha}-a_{1}^{\alpha}} \int_{0}^{1} \bigl(a_{2}^{\alpha}- \bigl((1-t)a_{2}^{\alpha}+tx^{\alpha}\bigr) \bigr) \bigl[(1-t)\big|h'(a_{2})\big| +t\big|h'(x)\big| \bigr]\,dt \\ \quad=\frac{x-a_{1}}{a_{2}^{\alpha}-a_{1}^{\alpha}}\triangle_{1}+\frac {a_{2}-x}{a_{2}^{\alpha}-a_{1}^{\alpha}} \triangle_{2}.\end{gathered} $$

 □

### Corollary 2.3

*Let* $x=(a_{1}+a_{2})/2$. *Then Theorem *2.2 *leads to*

$$\begin{gathered} \biggl\vert h \biggl(\frac{a_{1}+a_{2}}{2} \biggr)-\frac{\alpha}{a_{2}^{\alpha }-a_{1}^{\alpha}} \int_{a_{1}}^{a_{2}}h(s)\,d_{\alpha}s \biggr\vert \\ \quad\leq\frac{a_{2}-a_{1}}{2(a_{2}^{\alpha}-a_{1}^{\alpha})} \biggl[ \biggl(\frac {2a_{1}^{\alpha-1}a_{2}-10a_{1}^{\alpha}+a_{1} +a_{2}}{24} \biggr)\big|h'(a_{1})\big|+ \frac{a_{1}}{12} \biggl(\frac {a_{1}+a_{2}}{2} \biggr)^{\alpha-1}\big|h'(a_{1})\big| \\ \qquad{}+ \biggl(\frac{5a_{1}+3a_{2}-12a_{1}^{\alpha}}{24} \biggr) \bigg|h' \biggl(\frac {a_{1}+a_{2}}{2} \biggr) \bigg| +\frac{a_{1}}{12} \biggl(\frac{a_{1}+a_{2}}{2} \biggr)^{\alpha-1} \bigg|h' \biggl(\frac{a_{1}+a_{2}}{2} \biggr) \bigg| \\ \qquad{}+\frac{1}{6}a_{2}^{\alpha}\big|h'(a_{2})\big| -\frac{1}{6} \biggl(\frac{a_{1}+a_{2}}{2} \biggr)^{\alpha}\big|h'(a_{2})\big| \\ \qquad{}+\frac{a_{2}^{\alpha}}{3} \bigg|h' \biggl(\frac{a_{1}+a_{2}}{2} \biggr) \bigg| - \frac{1}{3} \biggl(\frac{a_{1}+a_{2}}{2} \biggr)^{\alpha} \bigg|h' \biggl(\frac {a_{1}+a_{2}}{2} \biggr) \bigg| \biggr].\end{gathered} $$

### Remark 2.4

If $\alpha=1$, then Corollary 2.3 becomes

$$ \begin{aligned} \biggl\vert h \biggl(\frac{a_{1}+a_{2}}{2} \biggr)-\frac{\alpha}{a_{2}^{\alpha }-a_{1}^{\alpha}} \int_{a_{1}}^{a_{2}}h(s)\,d_{\alpha}s \biggr\vert &\leq\frac{a_{2}-a_{1}}{24} \biggl(\big|h'(a_{1})\big|+4 \bigg|h' \biggl(\frac {a_{1}+a_{2}}{2} \biggr) \bigg|+\big|h'(a_{2})\big| \biggr) \\ &\leq\frac{a_{2}-a_{1}}{8} \bigl(\big|h'(a_{1})\big|+\big|h'(a_{2})\big| \bigr),\end{aligned} $$

where the second inequality is obtained by using the convexity of $|h'|$.

### Theorem 2.5

*Let* $q>1$, $M>0$, $0<\alpha\leq1$, $0\leq a_{1}< a_{2}$, $h: [a_{1}, a_{2}]\rightarrow\mathbb{R}$ *be an* *α*-*fractional differentiable function and* $D_{\alpha}(h)\in L_{\alpha}^{1}([a_{1}, a_{2}])$. *Then the inequality*

$$\begin{gathered} \bigg|h(x)-\frac{\alpha}{a_{2}^{\alpha}-a_{1}^{\alpha}} \int _{a_{1}}^{a_{2}}h(s)\,d_{\alpha}s \bigg| \\\quad\leq M\frac{x-a_{1}}{a_{2}^{\alpha}-a_{1}^{\alpha}} \bigl(A_{1}(\alpha) \bigr)^{1-1/q} \bigl(A_{2}(\alpha)+A_{3}(\alpha ) \bigr)^{1/q} \\\qquad{}+M\frac{a_{2}-x}{a_{2}^{\alpha}-a_{1}^{\alpha}} \bigl(B_{1}(\alpha) \bigr)^{1-1/q} \bigl(B_{2}(\alpha)+B_{3}(\alpha) \bigr)^{1/q}\end{gathered} $$

*holds if* $|h^{\prime}|^{q}$ *is convex on* $[a_{1}, a_{2}]$ *and* $|h^{\prime}(x)|^{q}\leq M$, *where*

$$\begin{gathered} A_{1}(\alpha)=\frac{x^{\alpha+1}-a_{1}^{\alpha+1}}{(\alpha +1)(x-a_{1})}-a_{1}^{\alpha}, \qquad B_{1}(\alpha)=a_{2}^{\alpha}-\frac {x^{\alpha+1}-a_{2}^{\alpha+1}}{(\alpha+1)(a_{2}-x)}, \\A_{2}(\alpha)=-\frac{a_{1}^{\alpha+1}}{(\alpha+1)(x-a_{1})}\frac{(\alpha +2)(x-a_{1})+a_{1}}{ (\alpha+2)(x-a_{1})}+ \frac{x^{\alpha+2}}{(\alpha+1)(x-a_{1})^{2}(\alpha +2)}-\frac{a_{1}^{\alpha}}{2}, \\B_{2}(\alpha)=\frac{a_{2}^{\alpha}}{2}+\frac{a_{2}^{\alpha+1}}{(\alpha +1)(a_{2}-x)}\frac{(\alpha+2)(a_{2}-x)+a_{2}}{ (\alpha+2)(a_{2}-x)}- \frac{x^{\alpha+2}}{(\alpha+1)(a_{2}-x)^{2}(\alpha+2)}, \\A_{3}(\alpha)=\frac{x^{\alpha+1}}{(\alpha+1)(x-a_{1})}\frac{(\alpha +2)(x-a_{1})-x}{(\alpha+2)(x-a_{1})} + \frac{a_{1}^{\alpha+2}}{(\alpha+1)(x-a_{1})^{2}(\alpha+2)}-\frac {a_{1}^{\alpha}}{2}, \\B_{3}(\alpha)=\frac{a_{2}^{\alpha}}{2}-\frac{x^{\alpha+1}}{(\alpha +1)(a_{2}-x)} \frac{(\alpha+2)(a_{2}-x)-x}{ (\alpha+2)(a_{2}-x)}-\frac{a_{2}^{\alpha+2}}{(\alpha +1)(a_{2}-x)^{2}(\alpha+2)}.\end{gathered} $$

### Proof

From Lemma 2.1, power-mean inequality and the convexity of $|h^{\prime }|^{q}$ together with the identities

$$ \int_{0}^{1} \bigl(\bigl((1-t)a_{1}+tx \bigr)^{\alpha}-a_{1}^{\alpha} \bigr)\,dt = \frac{x^{\alpha+1}-a_{1}^{\alpha+1}}{(\alpha+1)(x-a_{1})}-a_{1}^{\alpha} $$

and

$$ \int_{0}^{1} \bigl(a_{2}^{\alpha}- \bigl((1-t)a_{2}+tx\bigr)^{\alpha} \bigr)\,dt =a_{2}^{\alpha}- \frac{x^{\alpha+1}-a_{2}^{\alpha+1}}{(\alpha+1)(a_{2}-x)} $$

we clearly see that

2.1 $$\begin{aligned}& \begin{aligned}[t] &\biggl\vert h(x)-\frac{\alpha}{a_{2}^{\alpha}-a_{1}^{\alpha}} \int _{a_{1}}^{a_{2}}h(s)\,d_{\alpha}s \biggr\vert \\&\quad\leq\frac{x-a_{1}}{a_{2}^{\alpha}-a_{1}^{\alpha}} \int_{0}^{1} \bigl(\bigl((1-t)a_{1}+tx \bigr)^{\alpha}-a_{1}^{\alpha} \bigr) \big|h' \bigl((1-t)a_{1}+tx\bigr) \big|\,dt \\&\qquad+\frac{a_{2}-x}{a_{2}^{\alpha}-a_{1}^{\alpha}} \int_{0}^{1} \bigl(a_{2}^{\alpha}- \bigl((1-t)a_{2}+tx\bigr)^{\alpha} \bigr) \big|h' \bigl((1-t)a_{2}+tx\bigr) \big|\,dt,\end{aligned} \end{aligned}$$

2.2 $$\begin{aligned}& \begin{aligned}[t] &\int_{0}^{1} \bigl(\bigl((1-t)a_{1}+tx \bigr)^{\alpha}-a_{1}^{\alpha} \bigr) \big|h' \bigl((1-t)a_{1}+tx\bigr) \big|\,dt \\&\quad\leq \biggl( \int_{0}^{1} \bigl(\bigl((1-t)a_{1}+tx \bigr)^{\alpha}-a_{1}^{\alpha} \bigr)\,dt \biggr)^{1-1/q}\\ &\qquad\times \biggl( \int_{0}^{1} \bigl(\bigl((1-t)a_{1}+tx \bigr)^{\alpha}-a_{1}^{\alpha} \bigr) \big|h' \bigl((1-t)a_{1}+tx\bigr) \big|^{q}\,dt \biggr)^{1/q},\end{aligned} \end{aligned}$$

2.3 $$\begin{aligned}& \begin{aligned}[t] &\int_{0}^{1} \bigl(a_{2}^{\alpha}- \bigl((1-t)a_{2}+tx\bigr)^{\alpha} \bigr) \big|h' \bigl((1-t)a_{2}+tx\bigr) \big|\,dt \\&\quad\leq \biggl( \int_{0}^{1} \bigl( a_{2}^{\alpha}- \bigl((1-t)a_{2}+tx\bigr)^{\alpha} \bigr)\,dt \biggr)^{1-1/q}\\ &\qquad\times \biggl( \int_{0}^{1} \bigl(a_{2}^{\alpha}- \bigl((1-t)a_{2}+tx\bigr)^{\alpha} \bigr) \bigl\vert h'\bigl((1-t)a_{2}+tx\bigr) \bigr\vert ^{q}\,dt \biggr)^{1/q},\end{aligned} \end{aligned}$$

2.4 $$\begin{aligned}& \begin{aligned}[t] &\int_{0}^{1} \bigl(\bigl((1-t)a_{1}+tx \bigr)^{\alpha}-a_{1}^{\alpha} \bigr) \bigl\vert h'\bigl((1-t)a_{1}+tx\bigr) \bigr\vert ^{q}\,dt \\&\quad\leq \int_{0}^{1} \bigl(\bigl((1-t)a_{1}+tx \bigr)^{\alpha}-a_{1}^{\alpha} \bigr) \bigl[(1-t)\big|h'(a_{1})\big|^{q}+t\big|h'(x)\big|^{q} \bigr]\,dt \\&\quad=\big|h'(a)\big|^{q} \int_{0}^{1} \bigl(\bigl((1-t)a_{1}+tx \bigr)^{\alpha}-a_{1}^{\alpha} \bigr) (1-t)\,dt+\big|h'(x)\big|^{q} \int_{0}^{1} \bigl(\bigl((1-t)a_{1}+tx \bigr)^{\alpha}-a_{1}^{\alpha} \bigr)t \,dt \\&\quad=\big|h'(a)\big|^{q} \biggl(-\frac{a_{1}^{\alpha+1}}{(\alpha+1)(x-a_{1})} \frac{(\alpha +2)(x-a_{1})+a_{1}}{ (\alpha+2)(x-a_{1})}+\frac{x^{\alpha+2}}{(\alpha+1)(x-a_{1})^{2}(\alpha +2)}-\frac{a_{1}^{\alpha}}{2} \biggr) \\&\qquad+\big|h'(x)\big|^{q} \biggl(\frac{x^{\alpha+1}}{(\alpha+1)(x-a_{1})} \frac{(\alpha +2)(x-a_{1})-x}{ (\alpha+2)(x-a_{1})}+\frac{a_{1}^{\alpha+2}}{(\alpha+1)(x-a_{1})^{2}(\alpha +2)}-\frac{a_{1}^{\alpha}}{2} \biggr) \\&\quad\leq M^{q} \biggl(-\frac{a_{1}^{\alpha+1}}{(\alpha+1)(x-a_{1})}\frac{(\alpha +2)(x-a_{1})+a_{1}}{(\alpha+2)(x-a_{1})} + \frac{x^{\alpha+2}}{(\alpha+1)(x-a_{1})^{2}(\alpha+2)}-\frac {a_{1}^{\alpha}}{2} \biggr) \\&\qquad+M^{q} \biggl(\frac{x^{\alpha+1}}{(\alpha+1)(x-a_{1})}\frac{(\alpha +2)(x-a_{1})-x}{(\alpha+2)(x-a_{1})} + \frac{a_{1}^{\alpha+2}}{(\alpha+1)(x-a_{1})^{2}(\alpha+2)}-\frac {a_{1}^{\alpha}}{2} \biggr),\end{aligned} \end{aligned}$$

2.5 $$\begin{aligned}& \begin{aligned}[t] &\int_{0}^{1} \bigl(a_{2}^{\alpha}- \bigl((1-t)a_{2}+tx\bigr)^{\alpha} \bigr) \bigl\vert h'\bigl((1-t)a_{2}+tx\bigr) \bigr\vert ^{q}\,dt \\&\quad\leq \int_{0}^{1} \bigl(a_{2}^{\alpha}- \bigl((1-t)a_{2}+tx\bigr)^{\alpha} \bigr) \bigl[(1-t)\big|h'(a_{2})\big|^{q}+t\big|h'(x)\big|^{q} \bigr]\,dt \\&\quad=\big|h'(a_{2})\big|^{q} \int_{0}^{1} \bigl(a_{2}^{\alpha}- \bigl((1-t)a_{2}+tx\bigr)^{\alpha } \bigr) (1-t)\,dt\\ &\qquad +\big|h'(x)\big|^{q} \int_{0}^{1} \bigl(a_{2}^{\alpha}- \bigl((1-t)a_{2}+tx\bigr)^{\alpha} \bigr)t \,dt \\&\quad=\big|h'(a_{2})\big|^{q} \biggl(\frac{a_{2}^{\alpha}}{2}+ \frac{a_{2}^{\alpha +1}}{(\alpha+1)(a_{2}-x)} \frac{(\alpha+2)(a_{2}-x)+a_{2}}{(\alpha+2)(a_{2}-x)}-\frac{x^{\alpha +2}}{(\alpha+1)(a_{2}-x)^{2}(\alpha+2)} \biggr) \\&\qquad+\big|h'(x)\big|^{q} \biggl(\frac{a_{2}^{\alpha}}{2}- \frac{x^{\alpha+1}}{(\alpha +1)(a_{2}-x)} \frac{(\alpha+2)(a_{2}-x)-x}{(\alpha+2)(a_{2}-x)}-\frac{a_{2}^{\alpha+2}}{ (\alpha+1)(a_{2}-x)^{2}(\alpha+2)} \biggr) \\&\quad\leq M^{q} \biggl(\frac{a_{2}^{\alpha}}{2}+\frac{a_{2}^{\alpha+1}}{(\alpha +1)(a_{2}-x)} \frac{(\alpha+2)(a_{2}-x)+a_{2}}{(\alpha+2)(a_{2}-x)}-\frac{x^{\alpha +2}}{(\alpha+1)(a_{2}-x)^{2}(\alpha+2)} \biggr) \\&\qquad+M^{q} \biggl(\frac{a_{2}^{\alpha}}{2}-\frac{x^{\alpha+1}}{(\alpha+1)(a_{2}-x)} \frac{(\alpha+2)(a_{2}-x)-x}{(\alpha+2)(a_{2}-x)}-\frac{a_{2}^{\alpha+2}}{ (\alpha+1)(a_{2}-x)^{2}(\alpha+2)} \biggr).\end{aligned} \end{aligned}$$

Therefore, Theorem 2.5 follows easily from (2.1)–(2.5). □

### Remark 2.6

Let $\alpha=1$. Then Theorem 2.5 leads to

$$\begin{aligned} \bigg|h(x)-\frac{1}{a_{2}-a_{1}} \int_{a_{1}}^{a_{2}}h(s)\,ds \bigg| \leq{}& M\frac{x-a_{1}}{a_{2}-a_{1}} \bigl(A_{1}(1) \bigr)^{1-1/q} \bigl[A_{2}(1)+ A_{3}(1) \bigr]^{1/q} \\&+M\frac{a_{2}-x}{a_{2}-a_{1}} \bigl(B_{1}(1) \bigr)^{1-1/q} \bigl[B_{2}(1)+B_{3}(1) \bigr]^{1/q},\end{aligned} $$

where

$$\begin{gathered} A_{1}(1)=\frac{x-a_{1}}{2}, \qquad B_{1}(1)= \frac{a_{2}-x}{2}, \\ A_{2}(1)=\frac {3a_{1}^{2}x+6a_{1}^{2}+x^{3}-3a_{1}x^{2}-3a_{1}^{3}}{6(x-a_{1})^{2}}, \qquad B_{2}(1)= \frac{7a_{2}^{3}+3a_{2}x^{2}-9a_{2}^{2}x-x^{3}}{6(a_{2}-x)^{2}}, \\ A_{3}(1)=\frac{2x^{3}-2a_{1}^{3}-6a_{1}x^{2}+6a_{1}^{2}x}{6(x-a_{1})^{2}}, \qquad B_{3}(1)= \frac{2a_{2}^{3}-6a_{2}x+2x^{3}}{6(a_{2}-x)^{2}}.\end{gathered} $$

### Theorem 2.7

*Let* $q>1$, $M>0$, $0<\alpha\leq1$, $0\leq a_{1}< a_{2}$, $h: [a_{1}, a_{2}]\rightarrow\mathbb{R}$ *be an* *α*-*fractional differentiable function and* $D_{\alpha}(h)\in L_{\alpha}^{1}([a_{1}, a_{2}])$. *Then the inequality*

$$\begin{gathered} \bigg|h(x)-\frac{\alpha}{a_{2}^{\alpha}-a_{1}^{\alpha}} \int _{a_{1}}^{a_{2}}h(s)\,d_{\alpha}s \bigg| \\ \quad\leq M\frac{x-a_{1}}{a_{2}^{\alpha}-a_{1}^{\alpha}} \bigl(A_{1}(\alpha) \bigr)^{1-1/q} \biggl(\frac{-8a_{1}^{\alpha }+2a_{1}^{\alpha-1}x+2x^{\alpha-1}a_{1}+4x^{\alpha}}{12} \biggr)^{1/q} \\ \qquad{}+M\frac{a_{2}-x}{a_{2}^{\alpha}-a_{1}^{\alpha}} \bigl(B_{1}(\alpha) \bigr)^{1-1/q} \biggl(\frac{a_{2}^{\alpha}-x^{\alpha}}{2} \biggr)^{1/q}\end{gathered} $$

*holds if* $|h^{\prime}|^{q}$ *is convex on* $[a_{1}, a_{2}]$ *and* $|h^{\prime}(x)|^{q}\leq M$, *where*

$$ A_{1}(\alpha)=\frac{2a_{1}^{\alpha}+a_{1}^{\alpha-1}x+x^{\alpha -1}a_{1}+2x^{\alpha}-6a_{1}^{\alpha}}{6},\qquad B_{1}(\alpha)= \frac {a_{2}^{\alpha}-x^{\alpha}}{2}. $$

### Proof

It follows from the proof of Theorem 2.2 that

2.6 $$ \begin{aligned}[t] &\biggl\vert h(x)-\frac{\alpha}{a_{2}^{\alpha}-a_{1}^{\alpha}} \int _{a_{1}}^{a_{2}}h(s)\,d_{\alpha}s \biggr\vert \\ &\quad\leq\frac{x-a_{1}}{a_{2}^{\alpha}-a_{1}^{\alpha}} \int_{0}^{1} \bigl((1-t)a_{1}^{\alpha-1}+tx^{\alpha-1} \bigr) \bigl(\bigl((1-t)a_{1}+tx\bigr)-a_{1}^{\alpha} \bigr)\big|h'\bigl((1-t)a_{1}+tx\bigr)\big|\,dt \\ &\qquad+\frac{a_{2}-x}{a_{2}^{\alpha}-a_{1}^{\alpha}} \int_{0}^{1} \bigl(a_{2}^{\alpha}- \bigl((1-t)a_{2}^{\alpha}+tx^{\alpha}\bigr) \bigr)\big|h'\bigl((1-t)a_{2}+tx\bigr)\big|\,dt.\end{aligned} $$

From the power-mean inequality and convexity of $|h^{\prime}|^{q}$ together with the identities

$$\int^{1}_{0}\bigl((1-t)a_{1}^{\alpha-1}+tx^{\alpha -1} \bigr) \bigl(\bigl((1-t)a_{1}+tx\bigr)-a_{1}^{\alpha} \bigr)\,dt =\frac{2a_{1}^{\alpha}+a_{1}^{\alpha-1}x+x^{\alpha-1}a_{1}+2x^{\alpha }-6a_{1}^{\alpha}}{6}$$

and

$$ \int_{0}^{1} \bigl(a_{2}^{\alpha}- \bigl((1-t)a_{2}^{\alpha}+tx^{\alpha}\bigr) \bigr)\,dt= \frac{a_{2}^{\alpha}-x^{\alpha}}{2} $$

we get

2.7 $$\begin{aligned}& \begin{aligned}[t] &\int_{0}^{1} \bigl((1-t)a_{1}^{\alpha-1}+tx^{\alpha -1} \bigr) \bigl(\bigl((1-t)a_{1}+tx\bigr)-a_{1}^{\alpha} \bigr)\big|h'\bigl((1-t)a_{1}+tx\bigr)\big|\,dt \\ &\quad\leq \biggl( \int_{0}^{1} \bigl((1-t)a_{1}^{\alpha-1}+tx^{\alpha -1} \bigr) \bigl(\bigl((1-t)a_{1}+tx\bigr)-a_{1}^{\alpha} \bigr)\,dt \biggr)^{1-1/q} \\ &\qquad\times \biggl( \int_{0}^{1} \bigl((1-t)a_{1}^{\alpha-1}+tx^{\alpha -1} \bigr) \bigl(\bigl((1-t)a_{1}+tx\bigr)-a_{1}^{\alpha} \bigr)\big|h'\bigl((1-t)a_{1}+tx\bigr)\big|^{q}\,dt \biggr)^{1/q}, \end{aligned} \end{aligned}$$

2.8 $$\begin{aligned}& \begin{aligned}[t] &\int_{0}^{1} \bigl(a_{2}^{\alpha}- \bigl((1-t)a_{2}^{\alpha}+tx^{\alpha}\bigr) \bigr) \big|h'\bigl((1-t)a_{2}+tx\bigr) \big|\,dt \\&\quad\leq \biggl( \int_{0}^{1} \bigl( a_{2}^{\alpha}- \bigl((1-t)a_{2}^{\alpha }+tx^{\alpha}\bigr) \bigr)\,dt \biggr)^{1-1/q} \\&\qquad\times \biggl( \int_{0}^{1} \bigl(a_{2}^{\alpha}- \bigl((1-t)a_{2}^{\alpha }+tx^{\alpha}\bigr) \bigr) \bigl\vert h'\bigl((1-t)a_{2}+tx\bigr) \bigr\vert ^{q}\,dt \biggr)^{1/q},\end{aligned} \end{aligned}$$

2.9 $$\begin{aligned}& \begin{aligned}[t] &\int_{0}^{1} \bigl((1-t)a_{1}^{\alpha-1}+tx^{\alpha -1} \bigr) \bigl(\bigl((1-t)a_{1}+tx\bigr)-a_{1}^{\alpha} \bigr)\big|h'\bigl((1-t)a_{1}+tx\bigr)\big|^{q}\,dt \\&\quad\leq \int_{0}^{1} \bigl((1-t)a_{1}^{\alpha-1}+tx^{\alpha -1} \bigr) \bigl(\bigl((1-t)a_{1}+tx\bigr)-a_{1}^{\alpha} \bigr) \bigl[(1-t)\big|h'(a_{1})\big|^{q}+t\big|h'(x)\big|^{q} \bigr]\,dt \\&\quad=\big|h'(a_{1})\big|^{q} \int_{0}^{1}\bigl((1-t)a_{1}^{\alpha-1}+tx^{\alpha -1} \bigr) \bigl(\bigl((1-t)a_{1}+tx\bigr)-a_{1}^{\alpha} \bigr) (1-t)\,dt \\&\qquad+\big|h'(x)\big|^{q} \int_{0}^{1}\bigl((1-t)a_{1}^{\alpha-1}+tx^{\alpha -1} \bigr) \bigl(\bigl((1-t)a_{1}+tx\bigr)-a_{1}^{\alpha} \bigr)t\,dt \\&\quad=\big|h'(a_{1})\big|^{q} \biggl( \frac{1}{4}a_{1}^{\alpha}+\frac{1}{12}a_{1}^{\alpha -1}x+ \frac{1}{12}x^{\alpha-1}a_{1}+\frac{1}{12}x^{\alpha}- \frac {1}{2}a_{1}^{\alpha} \biggr) \\&\qquad+\big|h'(x)\big|^{q} \biggl(\frac{1}{12}a_{1}^{\alpha}+ \frac{1}{12}a_{1}^{\alpha -1}x+\frac{1}{12}x^{\alpha-1}a_{1}+ \frac{1}{4}x^{\alpha}-\frac {1}{2}a_{1}^{\alpha} \biggr) \\&\quad\leq M^{q} \biggl(\frac{-8a_{1}^{\alpha}+2a_{1}^{\alpha-1}x+2x^{\alpha -1}a_{1}+4x^{\alpha}}{12} \biggr),\end{aligned} \end{aligned}$$

2.10 $$\begin{aligned}& \begin{aligned}[t] &\int_{0}^{1} \bigl(a_{2}^{\alpha}- \bigl((1-t)a_{2}^{\alpha}+tx^{\alpha}\bigr) \bigr) \bigl\vert h'\bigl((1-t)a_{2}+tx\bigr) \bigr\vert ^{q}\,dt \\&\quad\leq \int_{0}^{1} \bigl(a_{2}^{\alpha}- \bigl((1-t)a_{2}^{\alpha}+tx^{\alpha }\bigr) \bigr) \bigl[(1-t)\big|h'(a_{2})\big|^{q}+t\big|h'(x)\big|^{q} \bigr]\,dt \\&\quad=\big|h'(a_{2})\big|^{q} \biggl(\frac{a_{2}^{\alpha}-x^{\alpha}}{6} \biggr)+\big|h'(x)\big|^{q} \biggl(\frac{a_{2}^{\alpha}-x^{\alpha}}{3} \biggr) \\&\quad\leq M^{q} \biggl(\frac{a_{2}^{\alpha}-x^{\alpha}}{2} \biggr).\end{aligned} \end{aligned}$$

Therefore, Theorem 2.7 follows easily from (2.6)–(2.10). □

### Theorem 2.8

*Let* $q>1$, $0<\alpha\leq1$, $0\leq a_{1}< a_{2}$, $h: [a_{1}, a_{2}]\rightarrow\mathbb{R}$ *be an* *α*-*fractional differentiable function and* $D_{\alpha}(h)\in L_{\alpha}^{1}([a_{1}, a_{2}])$. *Then the inequality*

$$\begin{gathered} \bigg|h(x)-\frac{\alpha}{a_{2}^{\alpha}-a_{1}^{\alpha}} \int _{a_{1}}^{a_{2}}h(s)\,d_{\alpha}s \bigg| \\\quad\leq\frac{x-a_{1}}{a_{2}^{\alpha}-a_{1}^{\alpha}}A_{1}(\alpha) \bigg|h' \biggl( \frac{C_{1}(\alpha)}{A_{1}(\alpha)} \biggr) \bigg| +\frac{a_{2}-x}{a_{2}^{\alpha}-a_{1}^{\alpha}}B_{1}(\alpha) \bigg|h' \biggl(\frac{C_{2}(\alpha)}{B_{1}(\alpha)} \biggr) \bigg|\end{gathered} $$

*holds if* $|h^{\prime}|^{q}$ *is concave on* $[a_{1}, a_{2}]$, *where*

$$\begin{aligned}& A_{1}(\alpha)=\frac{x^{\alpha+1}-a_{1}^{\alpha+1}}{(\alpha +1)(x-a_{1})}-a_{1}^{\alpha},\\& B_{1}(\alpha)=a_{2}^{\alpha}-\frac{x^{\alpha+1}-a_{2}^{\alpha +1}}{(\alpha+1)(a_{2}-x)}, \\& \begin{aligned}C_{1}(\alpha)={}&\frac{x^{\alpha+2}-a_{1}^{\alpha+2}}{(\alpha +1)(x-a_{1})}-\frac{x^{\alpha+3}+a_{1}^{\alpha+3}}{(\alpha +1)(x-a_{1})^{2}(\alpha+2)} \\&+\frac{a_{1}x}{(\alpha+1)(x-a_{1})^{2}(\alpha+2)} \bigl(x^{\alpha +1}+a_{1}^{\alpha+1} \bigr)-a_{1}^{\alpha}\frac{(a_{1}+x)}{2},\end{aligned} \\& \begin{aligned}C_{2}(\alpha)={}&a_{2}^{\alpha}\frac{(a_{2}+x)}{2}+ \frac{a_{2}^{\alpha +3}+x^{\alpha+3}}{(\alpha+1)(a_{2}-x)^{2}(\alpha+2)} \\&-\frac{a_{2}x}{(\alpha+1)(a_{2}-x)^{2}(\alpha+2)} \bigl(x^{\alpha +1}+a_{2}^{\alpha+1} \bigr)+\frac{a_{2}^{\alpha+2}-x^{\alpha+2}}{(\alpha +1)(a_{2}-x)}.\end{aligned} \end{aligned}$$

### Proof

It is well known that $|h^{\prime}|$ is concave due to $|h^{\prime }|^{q}$ being concave. It follows from Lemma 2.1 that

$$\begin{gathered} \bigg|h(x)-\frac{\alpha}{a_{2}^{\alpha}-a_{1}^{\alpha}} \int _{a_{1}}^{a_{2}}h(s)\,d_{\alpha}s \bigg| \\\quad\leq\frac{x-a_{1}}{a_{2}^{\alpha}-a_{1}^{\alpha}} \int_{0}^{1} \bigl(\bigl((1-t)a_{1}+tx \bigr)^{\alpha}-a_{1}^{\alpha} \bigr)\big|h' \bigl((1-t)a_{1}+tx\bigr)\big|\,dt \\\qquad{}+\frac{a_{2}-x}{a_{2}^{\alpha}-a_{1}^{\alpha}} \int_{0}^{1} \bigl(a_{2}^{\alpha}- \bigl((1-t)a_{2}+tx\bigr)^{\alpha} \bigr)\big|h' \bigl((1-t)a_{2}+tx\bigr)\big|\,dt.\end{gathered} $$

Making use of Jensen’s integral inequality, we have

$$\begin{gathered} \int_{0}^{1} \bigl(\bigl((1-t)a_{1}+tx \bigr)^{\alpha}-a_{1}^{\alpha} \bigr) \bigl\vert h'\bigl((1-t)a_{1}+tx\bigr) \bigr\vert \,dt \\\quad\leq \int_{0}^{1} \bigl(\bigl((1-t)a_{1}+tx \bigr)^{\alpha}-a_{1}^{\alpha} \bigr) \bigg|h' \biggl(\frac{\int_{0}^{1} (((1-t)a_{1}+tx)^{\alpha}-a_{1}^{\alpha } )((1-t)a_{1}+tx)\,dt}{ \int_{0}^{1} (((1-t)a_{1}+tx)^{\alpha}-a_{1}^{\alpha} )\,dt} \biggr) \bigg|\,dt \\\quad=A_{1}(\alpha) \bigg|h' \biggl(\frac{C_{1}(\alpha)}{A_{1}(\alpha)} \biggr) \bigg|, \\\int_{0}^{1} \bigl(a_{2}^{\alpha}- \bigl((1-t)a_{2}+tx\bigr)^{\alpha} \bigr) \bigl\vert h'\bigl((1-t)a_{2}+tx\bigr) \bigr\vert \,dt \\\quad\leq \int_{0}^{1} \bigl(a_{2}^{\alpha}- \bigl((1-t)a_{2}+tx\bigr)^{\alpha} \bigr) \bigg|h' \biggl( \frac{\int_{0}^{1} (a_{2}^{\alpha }-((1-t)a_{2}+tx)^{\alpha} )((1-t)a_{2}+tx)\,dt}{ \int_{0}^{1} (a_{2}^{\alpha}-((1-t)a_{2}+tx)^{\alpha} )\,dt} \biggr) \bigg|\,dt \\\quad=B_{1}(\alpha) \bigg|h' \biggl(\frac{C_{2}(\alpha)}{B_{1}(\alpha)} \biggr)\bigg|,\end{gathered} $$

where we have used the identities

$$\begin{gathered} \int_{0}^{1} \bigl(\bigl((1-t)a_{1}+tx \bigr)^{\alpha}-a_{1}^{\alpha} \bigr)\,dt=A_{1}( \alpha ), \\ \int_{0}^{1} \bigl(a_{2}^{\alpha}- \bigl((1-t)a_{2}+tx\bigr)^{\alpha} \bigr)\,dt =B_{1}( \alpha), \\\int_{0}^{1} \bigl(\bigl((1-t)a_{1}+tx \bigr)^{\alpha}-a_{1}^{\alpha} \bigr) \bigl((1-t)a_{1}+tx \bigr)\,dt=C_{1}(\alpha), \\\int_{0}^{1} \bigl(a_{2}^{\alpha}- \bigl((1-t)x+ta_{2}\bigr)^{\alpha} \bigr) \bigl((1-t)a_{2}+tx \bigr)\,dt=C_{2}(\alpha).\end{gathered} $$

 □

### Remark 2.9

If $\alpha=1$, then Theorem 2.8 becomes

$$\begin{gathered} \bigg|h(x)-\frac{\alpha}{a_{2}^{\alpha}-a_{1}^{\alpha}} \int _{a_{1}}^{a_{2}}h(s)\,d_{\alpha}s \bigg| \\\quad\leq\frac{(x-a_{1})^{2}}{2(a_{2}-a_{1})} \bigg|h' \biggl(\frac {2x^{4}-5a_{1}x^{3}+3a_{1}^{2}x^{2}+xa_{1}^{3}-a_{1}^{4}}{3(x-a_{1})} \biggr) \bigg| \\\qquad{}+\frac{(a_{2}-x)^{2}}{2(a_{2}-a_{1})} \bigg|h' \biggl(\frac {4x^{4}-a_{2}x^{3}-3x^{2}a_{2}^{2}-7a_{2}^{3}x+7a_{2}^{4}}{3(a_{2}-x)} \biggr) \bigg|.\end{gathered} $$

### Theorem 2.10

*Let* $q>1$, $0<\alpha\leq1$, $0\leq a_{1}< a_{2}$, $h: [a_{1}, a_{2}]\rightarrow\mathbb{R}$ *be an* *α*-*fractional differentiable function and* $D_{\alpha}(h)\in L_{\alpha}^{1}([a_{1}, a_{2}])$. *Then the inequality*

$$\begin{gathered} \bigg|h(x)-\frac{\alpha}{a_{2}^{\alpha}-a_{1}^{\alpha}} \int _{a_{1}}^{a_{2}}h(s)\,d_{\alpha}s \bigg| \\\quad\leq\frac{x-a_{1}}{a_{2}^{\alpha}-a_{1}^{\alpha}}A_{1}(\alpha) \bigg|h' \biggl( \frac{C_{1}(\alpha)}{A_{1}(\alpha)} \biggr) \bigg| +\frac{a_{2}-x}{a_{2}^{\alpha}-a_{1}^{\alpha}}B_{1}(\alpha) \bigg|h' \biggl(\frac{C_{2}(\alpha)}{B_{1}(\alpha)} \biggr) \bigg|\end{gathered} $$

*holds if* $|h^{\prime}|^{q}$ *is concave on* $[a_{1}, a_{2}]$, *where*

$$\begin{gathered} A_{1}(\alpha)=\frac{2a_{1}^{\alpha}+a_{1}^{\alpha-1}x+x^{\alpha -1}a_{1}+2x^{\alpha}-6a_{1}^{\alpha}}{6},\qquad B_{1}(\alpha)= \frac {a_{2}^{\alpha}-x^{\alpha}}{2}, \\C_{1}(\alpha)=\frac{-3a_{1}^{\alpha+1}+x+x^{\alpha-1}a_{1}^{\alpha +1}+x^{\alpha}a_{1}-5xa_{1}^{\alpha}+x^{2}a_{1}^{\alpha-1}+x a_{1}+3x^{\alpha+1}}{12}, \\C_{2}(\alpha)=\frac{a_{2}^{\alpha+1}-x^{\alpha}a_{2}+2xa_{2}^{\alpha }-2x^{\alpha+1}}{6}.\end{gathered} $$

### Proof

From the concavity of $|h^{\prime}|^{q}$ we know that $|h^{\prime}|$ is also concave, then from Lemma 2.1 we have

$$\begin{gathered} \biggl\vert h(x)-\frac{\alpha}{a_{2}^{\alpha}-a_{1}^{\alpha}} \int _{a_{1}}^{a_{2}}h(s)\,d_{\alpha}s \biggr\vert \\\quad\leq\frac{x-a_{1}}{a_{2}^{\alpha}-a_{1}^{\alpha}} \int _{0}^{1}\bigl((1-t)a_{1}^{\alpha-1}+tx^{\alpha-1} \bigr) \bigl(\bigl((1-t)a_{1}+tx\bigr)-a_{1}^{\alpha } \bigr)\big|h'\bigl((1-t)a_{1}+tx\bigr)\big|\,dt \\\qquad{}+\frac{a_{2}-x}{a_{2}^{\alpha}-a_{1}^{\alpha}} \int_{0}^{1} \bigl(a_{2}^{\alpha}- \bigl((1-t)a_{2}^{\alpha}+tx^{\alpha}\bigr) \bigr)\big|h'\bigl((1-t)a_{2}+tx\bigr)\big|\,dt.\end{gathered} $$

It follows from the Jensen integral inequality that

$$\begin{aligned}& \int_{0}^{1}\bigl((1-t)a_{1}^{\alpha-1}+tx^{\alpha -1} \bigr) \bigl(\bigl((1-t)a_{1}+tx\bigr)-a_{1}^{\alpha} \bigr)\big|h'\bigl((1-t)a_{1}+tx\bigr)\big|\,dt \\& \quad\leq \int_{0}^{1}\bigl((1-t)a_{1}^{\alpha-1}+tx^{\alpha -1} \bigr) \bigl(\bigl((1-t)a_{1}+tx\bigr)-a_{1}^{\alpha} \bigr) \\& \qquad{}\times \bigg|h' \biggl(\frac{\int_{0}^{1}((1-t)a_{1}^{\alpha-1}+tx^{\alpha -1})(((1-t)a_{1}+tx)-a_{1}^{\alpha})((1-t)a_{1}+tx)\,dt}{ \int_{0}^{1}((1-t)a_{1}^{\alpha-1}+tx^{\alpha -1})(((1-t)a_{1}+tx)-a_{1}^{\alpha})\,dt} \biggr)\bigg|\,dt \\& \quad=A_{1}(\alpha)h' \biggl(\frac{C_{1}(\alpha)}{A_{1}(\alpha)} \biggr), \\& \int_{0}^{1} \bigl(a_{2}^{\alpha}- \bigl((1-t)a_{2}^{\alpha}+tx^{\alpha}\bigr) \bigr) \bigl\vert h'\bigl((1-t)a_{2}+tx\bigr) \bigr\vert \,dt \\& \quad\leq \int_{0}^{1} \bigl(a_{2}^{\alpha}- \bigl((1-t)a_{2}^{\alpha}+tx^{\alpha }\bigr) \bigr) \bigg|h' \biggl(\frac{\int_{0}^{1} (a_{2}^{\alpha}-((1-t)a_{2}^{\alpha }+tx^{\alpha}) )((1-t)a_{2}+tx)\,dt}{ \int_{0}^{1} (a_{2}^{\alpha}-((1-t)a_{2}^{\alpha}+tx^{\alpha }) )\,dt} \biggr) \bigg| \\& \quad=B_{1}(\alpha)h' \biggl(\frac{C_{2}(\alpha)}{B_{1}(\alpha)} \biggr). \end{aligned}$$

 □

### Remark 2.11

If $\alpha=1$, then Theorem 2.10 leads to

$$\begin{gathered} \bigg|h(x)-\frac{1}{a_{2}-a_{1}} \int_{a_{1}}^{a_{2}}h(s)\,ds \bigg| \\\quad\leq\frac{(x-a_{1})^{2}}{2(a_{2}-a_{1})} \bigg|h' \biggl(\frac {2x^{2}-a_{1}x-a_{1}^{2}}{3(x-a_{1})} \biggr) \bigg| + \frac{(a_{2}-x)^{2}}{2(a_{2}-a_{1})} \bigg|h' \biggl(\frac {a_{2}^{2}+a_{2}x-2x^{2}}{3(a_{2}-x)} \biggr) \bigg|.\end{gathered} $$

### Remark 2.12

If $\alpha=1$ and $x=(a_{1}+a_{2})/2$, then Theorem 2.10 becomes

$$ \bigg|h(x)-\frac{1}{a_{2}-a_{1}} \int_{a_{1}}^{a_{2}}h(s)\,ds \bigg| \leq\frac{a_{2}-a_{1}}{8} \biggl[ \bigg|h' \biggl(\frac{2a_{1}+a_{2}}{3} \biggr) \bigg| + \bigg|h' \biggl(\frac{a_{1}+2a_{2}}{3} \biggr) \bigg| \biggr]. $$

## Applications to means

Let $a, b>0$ with $a\neq b$. Then the arithmetic mean $A(a, b)$, logarithmic mean $L(a,b)$ and generalized logarithmic mean $L_{(\alpha, r)}(a,b)$ of *a* and *b* are defined by

$$ A(a,b)=\frac{a+b}{2}, \qquad L(a,b)=\frac{b-a}{\log b-\log a}, \qquad L_{(\alpha, r)}(a,b)= \biggl[\frac{\alpha (b^{r+\alpha}-a^{r+\alpha } )}{(r+\alpha)(b^{\alpha}-a^{\alpha})} \biggr]^{1/r}, $$

respectively.

Recently, the bivariate means have been the subject of intensive research, many remarkable inequalities for the bivariate means can be found in the literature [25–60].

Let $h(x)=x^{r}$ and $h(x)=1/x$. Then Corollary 2.3 immediately leads to Theorems 3.1 and 3.2.

### Theorem 3.1

*Let* $r>1$ *and* $\alpha\in(0, 1]$. *Then the inequality*

$$\begin{gathered} \big|A^{r}(a_{1},a_{2})-L^{r}_{(\alpha,r)}(a_{1},a_{2})\big| \\\quad\leq\frac{r(a_{2}-a_{1})}{2(a_{2}^{\alpha}-a_{1}^{\alpha})} \biggl[ \biggl(\frac {2a_{1}^{\alpha-1}a_{2}-10a_{1}^{\alpha}+a_{1} +a_{2}}{24} \biggr)|a_{1}|^{r-1}+ \frac{a_{1}}{12} \biggl(\frac {a_{1}+a_{2}}{2} \biggr)^{\alpha-1}|a_{1}|^{r-1} \\\qquad{}+ \biggl(\frac{5a_{1}+3a_{2}-12a_{1}^{\alpha}}{24} \biggr) \bigg|\frac {a_{1}+a_{2}}{2} \bigg|^{r-1}+ \frac{a_{1}}{12} \biggl(\frac{a_{1}+a_{2}}{2} \biggr)^{\alpha-1} \bigg| \frac{a_{1}+a_{2}}{2} \bigg|^{r-1} \\\qquad{}+\frac{1}{6}a_{2}^{\alpha}|a_{2}|^{r-1}- \frac{1}{6} \biggl(\frac {a_{1}+a_{2}}{2} \biggr)^{\alpha}|a_{2}|^{r-1}+ \frac{a_{2}^{\alpha}}{3} \bigg|\frac{a_{1}+a_{2}}{2} \bigg|^{r-1} \\\qquad{}-\frac{1}{3} \biggl(\frac{a_{1}+a_{2}}{2} \biggr)^{\alpha} \bigg| \frac {a_{1}+a_{2}}{2} \bigg|^{r-1} \biggr]\end{gathered} $$

*holds for all* $a_{1}, a_{2}>0$.

### Theorem 3.2

*Let* $r>1$ *and* $\alpha\in(0, 1]$. *Then the inequality*

$$\begin{gathered} \big|A^{r}(a_{1},a_{2})-L^{r}_{(\alpha,r)}(a_{1},a_{2})\big| \\\quad\leq\frac{(a_{2}-a_{1})}{2(a_{2}^{\alpha}-a_{1}^{\alpha})} \biggl[ \biggl(\frac {2a_{1}^{\alpha-1}b-10a_{1}^{\alpha}+a_{1} +a_{2}}{24} \biggr)|a_{1}|^{-2}+ \frac{a_{1}}{12} \biggl(\frac {a_{1}+a_{2}}{2} \biggr)^{\alpha-1}|a_{1}|^{-2} \\\qquad{}+ \biggl(\frac{5a_{1}+3a_{2}-12a_{1}^{\alpha}}{24} \biggr) \bigg|\frac {a_{1}+a_{2}}{2} \bigg|^{-2}+ \frac{a_{1}}{12} \biggl(\frac{a_{1}+a_{2}}{2} \biggr)^{\alpha-1} \bigg| \frac {a_{1}+a_{2}}{2} \bigg|^{-2} \\\qquad{}+\frac{1}{6}a_{2}^{\alpha}|a_{2}|^{-2}- \frac{1}{6} \biggl(\frac {a_{1}+a_{2}}{2} \biggr)^{\alpha}|a_{2}|^{-2}+ \frac{a_{2}^{\alpha}}{3} \bigg|\frac{a_{1}+a_{2}}{2} \bigg|^{-2}\\ \qquad{}-\frac{1}{3} \biggl(\frac{a_{1}+a_{2}}{2} \biggr)^{\alpha} \bigg|\frac {a_{1}+a_{2}}{2} \bigg|^{-2} \biggr]\end{gathered} $$

*holds for all* $a_{1}, a_{2}>0$.

## Results and discussion

There are many results devoted to the well-known Ostrowski inequality. This inequality has many applications in the area of numerical analysis. In this paper, we give results for Ostrowski inequality containing conformable fractional integrals and their applications for means. First, we prove an identity associated with the Ostrowski inequality for conformable fractional integrals. By using this identity and convexity of different classes of functions and some well-known inequalities, we obtain several results for the inequality. The inequalities derived here are also pointed out to correspond to some known results, being special cases. At the end, we also present applications for means. The presented idea may stimulate further research in the theory of conformable fractional integrals.

## Conclusion

In this paper, we prove an identity associated with the Ostrowski inequality for conformable fractional integral, present several Ostrowski type inequalities involving the conformable fractional integrals, and provide the applications in bivariate means theory. The idea and results presented are novel and interesting.
